# A secondary analysis of a randomised controlled trial to investigate the effect of Tai Chi on the instrumented timed up and go test in people with mild to moderate dementia

**DOI:** 10.1007/s40520-020-01741-7

**Published:** 2020-11-03

**Authors:** Jonathan Williams, Samuel Nyman

**Affiliations:** 1 Department of Rehabilitation and Sport Sciences, Faculty of Health and Social Sciences, Bournemouth University, Poole, BH1 3LT UK; 2 Department of Psychology and Ageing & Dementia Research Centre, Faculty of Science and Technology, Bournemouth University, Poole, UK

**Keywords:** Balance, Gait, Turning, Sit to stand, Intervention, Clinical trial

## Abstract

**Background:**

Previous research has identified that Tai Chi is effective for reducing risk of falls and improving timed up and go scores. However, our previous research identified no-significant difference in time to complete the timed up and go test following a Tai Chi intervention in people with dementia.

**Aim:**

To conduct a secondary analysis to extend our understanding of the effect of Tai Chi on the instrumented Timed Up and Go test.

**Methods:**

This is a secondary analysis of a randomised controlled trial set in the community. People with dementia, recruited from NHS databases, memory clinics, local charities and self-referral across the south of England, received either 20 weeks of Tai Chi plus normal care or normal care. Outcomes were assessed using the instrumented Timed Up and Go test, completed at baseline and after 6 months.

**Results:**

From 83 people with dementia volunteering for the study, 67 complete datasets were available for analysis. Within-group pairwise comparison across time revealed no-significant gains for any of the instrumented Timed Up and Go variables, and no-significant difference for between-group pairwise comparisons.

**Discussion:**

This suggests that Tai Chi had no effect on the instrumented Timed Up and Go in people with dementia. This lack of effect may be due to the lack of specificity of the training stimulus to the outcome measure.

**Conclusion:**

Tai Chi had no effect on any instrumented Timed Up and Go variables, suggesting Tai Chi may not be best placed to enhance the sub-elements of the instrumented Timed Up and Go to reduce fall risk among community-dwelling people with dementia.

**Clinical trial registration number**: NCT02864056.

Falls among older people are globally recognised as a public health issue [1]. Falls in later life can result in injuries that require hospitalisation and reduce independence [2], and subsequently reduce quality of life and increase costs on health and social services [3]. A risk factor for falling is cognitive impairment, and in particular dementia, a degenerative neurological disease characterised by a chronic, global, and non-reversible loss of cognitive functioning [4]. People with dementia (PWD) are more than twice as likely to fall and twice as likely to experience injurious falls compared to their cognitively intact peers [5, 6].

There is robust evidence for interventions, and in particular exercise-based interventions, to prevent falls and fall-related injuries among community-dwelling people without cognitive impairment [7–9]. This includes Tai Chi exercise interventions: A meta-analysis found Tai Chi to reduce falls among the general healthy older adult population and those at risk of falls by on average 31% (incident rate ratio [IRR] = 0.69, 95% confidence interval [CI] 0.60, 0.80, 15 trials) and the number of people falling at least once by 20% (IRR = 0.80, 95% CI 0.72, 0.88, 16 trials) [12]. Furthermore, a meta-regression of 108 exercise trials with community-dwelling older people found that Tai Chi was one of three exercise programmes that are effective in reducing falls [13]. However, to date, only three exercise trials have been conducted with community dwelling PWD [10, 11, 14]. We recently conducted a randomised trial to test the effect of Tai Chi exercise on improving postural balance among PWD [15]. It was also a feasibility study for a subsequent definitive trial to test the effect of Tai Chi on preventing falls among PWD. Tai Chi is an ancient form of Chinese mind–body exercise, where participants carry out smooth and continuous body movements along with deep breathing and mental concentration [16], equivalent to moderate-intensity exercise and quiet meditation [17]. This form of exercise is particularly suited for PWD with its use of slow and repetitive movements [18].

The results of our recent trial suggested that PWD in the Tai Chi group, relative to a usual care control group, at 6-month follow-up had significantly greater scores for quality of life, and a strong trend for a reduction in falls [19]. However, these results were despite finding no difference between the Tai Chi and control group on measures of balance including the timed up and go test (primary outcome) [19]. The timed up and go (TUG) test requires participants to stand, walk 3 m, turn, walk back, and return to a seated position [20]. Such a test is well documented to predict fallers from non-fallers [21–23] as well as predict development of future dementia in a sample of over 49000 [24]. Despite this predictive ability, the overall measure of time to complete masks the individual subcomponents that, if isolated, could be analysed to identify early physical impairments [21]. Therefore, the instrumented timed up and go test (iTUG) has been proposed [22] and has been found to be a reliable and valid measure of physical performance [22–24]. Furthermore, the iTUG has demonstrated greater discriminatory ability than total time to complete TUG in those with mild cognitive impairment [21, 26, 27]. Such approaches were also able to provide specific insights into performance differences of those with a diagnosis of dementia compared to those without [28].

In light of the potential for the iTUG to reveal undetected physical improvements among PWD from practising Tai Chi, we conducted an ad hoc secondary analysis of the trial data. We hypothesised that PWD in the Tai Chi group would have superior scores on the iTUG at follow-up relative to the control group, and that these scores would be correlated with the observed trend for a reduction in falls in the Tai Chi group during the 6-month follow-up period relative to the control group.

## Methods

This study utilises data obtained as part of the TACIT trial (NTC02864056), a randomised controlled trial to investigate the impact of Tai Chi on balance in PWD and their informal carers. Ethical approval was granted by the West of Scotland Research Ethics Committee 4 (reference: 16/WS/0139 and the Health Research Authority (IRAS project ID: 209193). A detailed breakdown of the TACIT protocol has been previously published [15].

### Participants

PWD and their informal carers were recruited from NHS databases, local charities, memory clinics and through self-referral from around the South of England. To meet inclusion, PWD were aged 18 or over with a formal diagnosis of dementia (indicated by their NHS medical records), living at home and were able and willing to complete weekly standing Tai Chi without physical assistance. Exclusion criteria included being in receipt of palliative care, living in a care home, severe dementia (defined as 9 or less on the Mini-Addenbrooke’s Cognitive Evaluation) [29], a Lewy body dementia or dementia with Parkinson’s disease, severe sensory impairment, currently under the care of or have been referred to a falls clinic, or lacked mental capacity to provide informed consent. In addition, PWD were excluded if they were currently completing or had recently completed Tai Chi or similar.

### Randomisation

PWD were randomised using a centralised web-based randomisation system maintained by the UKCRC-registered Peninsula Clinical Trials Unit to either receive usual care plus Tai Chi or usual care (control group) in a 1:1 ratio. Minimisation was used within each site by treatment condition and 12-month fall history at baseline. All individuals involved with data collection were blinded to group allocation.

The sample size was based on that used for the Tacit trial [15]. The study was powered at 90% to achieve a difference of 4 s in total time to complete TUG, with a standard deviation of 0.38, a correlation of 0.7 and a two-sided 5% significance level. This yielded a target recruitment of 120. While the recruited sample was below target at 83 PWD and carers, smaller standard deviations than estimated were observed for the TUG and the estimated smallest detectable change of a value of 4 was outside the 95% confidence interval (− 2,17, 3.81) between the trial arms, suggesting that the testing on the TUG was adequately powered.

### Intervention

Usual care could include medications and support services, social groups, peer support but with an absence of exercise prescription. The intervention group continued usual care but added Tai Chi comprising of 3 elements: (1) Tai Chi classes, (2) home-based Tai Chi practice completed with carer and (3) behaviour change techniques (including action planning, coping planning, self-monitoring, feedback and social support) [15]. Classes were weekly and comprised of 45-min instructor-led Tai Chi followed by 45-min informal discussion over 20 weeks. Classes were held in a variety of suitable venues such as church halls. Home-based Tai Chi was based on repetition of the taught material with an aim to accrue 50-h practice. Tai Chi instructors were all experienced Tai Chi trainers and had qualifications to senior instructor level.

### Instrumentation

A miniature balance sensor device, housing an integration of triaxial accelerometer and triaxial gyroscope (THETAmetrix, Portsmouth, UK) sampling at 30 Hz was attached to the low back, reinforced with elasticated strap. The device provides data pertaining to linear accelerations and rotational velocities which was exported to MatLab where a bespoke algorithm determines the features of importance from the TUG. Details regarding the algorithm have been reported previously [30] and excellent reliability of the device has been determined [31]. Outcomes (listed in Table 2) relating to the sit-to-stand phase, the two walking phases and two turning phases are retrieved through the bespoke algorithm previously described [30].

All iTUG data were collected within the individual’s home and the iTUG comprised of a standard definition of stand from sitting, walk three metres, turn, walk back and sit down. One iTUG only was completed. No guidance was provided for direction of turn and the distance was marked with tape on the floor. A pragmatic approach to chair selection was used but every effort was made to complete the follow-up using the same chair. All iTUG data were collected without knowledge of group allocation. In addition to baseline iTUG performance, iTUG was repeated after 6 months post-baseline. In addition, to determine baseline function, a Berg balance scale was completed by the same individual [32].

### Statistics

All iTUG variables were not normally distributed; therefore, non-parametric statistics were used to explore differences. Between-group pairwise comparisons were made using Mann–Whitney U tests at baseline and at follow-up. In addition, pairwise comparisons were made using Wilcoxon tests, within group, across the two time points (baseline and follow-up) for both the control and intervention groups. A Bonferroni correction was applied to minimise the chance of type 2 error and thus, an alpha of 0.004 was used to determine statistical significance.

## Results

**Fig. 1:**
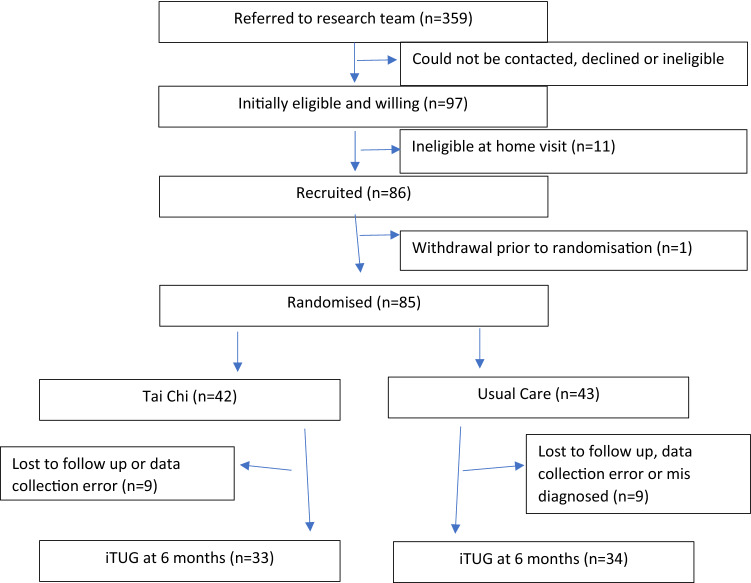
Flow diagram of study participation

Over the period of between 06/04/2017 and 17/07/2018, 359 individuals were approached with 85 agreeing to participate. Two individuals were erroneously diagnosed with dementia and were removed, from which data for 67 PWD were available at baseline and 6-month follow-up. 13 individuals were lost to follow-up and 3 individuals were removed due to data collection error (1 from intervention group and 2 from usual care group). This resulted in 33 for the intervention group and 34 for the control group, see Fig. 1 or [19]. No serious adverse events relating to participation were noted. There were no differences at baseline between the groups, including cognitive function (Table 1).

**Table 1:** Baseline characteristics between groups

	Intervention Group	Control Group
Female n (%)	14 (42%)	13 (38%)
Mean age (sd)	78.6 (8.4)	78.3 (8.0)
M-ACE (sd)	16.5 (5.2)	15.6 (4.5)
Berg Balance Scale	44.6 (5.9)	44.3 (7.5)

*M-ACE* mini-Addenbrooke’s cognitive evaluation

Baseline scores and score at six-month follow-up for iTUG for the 2 groups can be found in Table 2. Between-group pairwise comparisons demonstrated no significant differences between the intervention group and control group at both baseline and at follow-up for any iTUG variable. Within-group pairwise comparisons demonstrated that in the intervention group, there was a significant reduction in turning velocity for the second turn (p = 0.002) at follow-up, compared to baseline. No other significant differences were evident at follow-up in the intervention group. In the control group, there was a significant reduction in the turning velocity of the first turn (*p* = 0.003). No other significant difference was determined in the control group at follow-up.

**Table 2:** Instrumented Timed Up and Go variables at baseline and 6 months for the intervention and control groups

	Intervention Group		Control Group		Mean difference 95% CI
	Baseline Median (IQR)	Follow-up Median (IQR)	Baseline Median (IQR)	Follow-up Median (IQR)	
Standing Acc (ms^−2^)	− 1.56 (0.91)	− 1.38 (0.58)	− 1.54 (0.58)	− 1.50 (0.73)	0.01 (− 0.20, 0.22)
S2S duration (s)	2.16 (0.63)	2.10 (1.17)	2.03 (1.04)	1.94 (0.67)	− 0.33 (− 1.08, 0.41)
Walk 1 duration (s)	4.44 (2.25)	4.32 (3.08)	4.05 (2.25)	4.10 (2.10)	0.77 (− 0.40, 1.94)
Walk 2 duration (s)	3.81 (2.03)	3.49 (2.63)	3.51 (2.74)	3.71 (3.11)	0.28 (− 0.77, 1.32)
Turn 1 duration (s)	2.54 (0.79)	2.95 (0.73)	2.70 (0.69)	3.08 (0.67)	0.22 (− 0.55, 0.98)
Turn 1 Vel (^o^/s)	1.71 (0.62)	1.79 (0.40)	1.93 (0.81)	1.70 (0.57)*	− 0.04 (− 0.21, 0.12)
Turn 2 Vel (^o^/s)	1.80 (0.90)	1.67 (0.82)*	1.95 (0.91)	1.61 (1.23)	− 0.30 (− 0.49, − 0.10)
AC Step walk 1	0.63 (0.52)	0.59 (0.41)	0.63 (0.40)	0.59 (0.37)	− 0.38 (− 0.86, 0.11)
AC Stride walk 1	0.74 (0.54)	0.62 (0.54)	0.62 (0.43)	0.53 (0.42)	− 0.42 (− 0.88, 0.05)
Step/Stride Ratio 1	1.02 (0.17)	0.96 (0.34)	1.05 (0.50)	0.99 (0.40)	0.09 (− 0.22, 0.40)
AC Step walk 2	0.79 (0.42)	0.54 (0.47)	0.52 (0.46)	0.65 (0.49)	− 0.18 (− 0.37, 0.01)
AC Stride walk 2	0.79 (0.18)	0.69 (0.34)	0.68 (0.43)	0.66 (0.34)	− 0.20 (− 0.40, − 0.00)
Step/Stride Ratio 2	0.98 (0.30)	0.81 (0.37)	0.87 (0.42)	0.90 (0.49)	− 0.15 (− 0.32, 0.03)

*p < 0.004. IQR; interquartile range, *Acc* Acceleration, *S2S* sit to stand, *Vel* Velocity, *AC* Autocorrelation

## Discussion

The aim of this study was to explore the effects of Tai Chi on iTUG in people with dementia. Previously, it was identified that there was no significant difference in total time to complete the TUG [19] and this study adds to the understanding by demonstrating that this lack of effect is evident across all sub phases of the iTUG. This provides new comprehension, as each sub phase of the iTUG constitutes subtle or large differences in their underlying physiological constructs (i.e. quadriceps power for sit to stand, coordination for turning, etc.); however, despite this, none appeared to be modified by Tai Chi, suggesting a universal lack of treatment effect on iTUG.

These results are in conflict with other studies that have demonstrated significant enhancement in the total time to complete TUG following Tai Chi in older adults and individuals with Parkinsons Disease [33, 34].

The lack of effect may be explained by insufficient treatment dose. If the Tai Chi intervention lacked the magnitude and intensity to yield any physiological change, then this could possibly explain the lack of change demonstrated in the iTUG. Fidelity of the intervention has been reported previously [19], and all participants were able to understand and follow the Tai Chi instructions. The mean supervised Tai Chi practice time was 8.4 h, which is less than half of that offered by Zou et al. [35], who demonstrated significant reductions in total time to complete iTUG. However, the magnitude of change was less than 1 s on a baseline of 10.1 s, suggesting a minimal change on the background of minimal impairment, both of which are different to the current study. However, Hosseini et al. [36] delivered a similar amount of supervised Tai Chi to the current study which resulted in a 6.7 s improvement in total time to complete iTUG using a sample with a baseline score similar to the current study. It is not clear if the intensity was different and, thus, the Tai Chi more effective or whether the presence of cognitive impairment in our sample of PWD could explain the difference in the studies. The current study also included an additional mean of 16.5 h of Tai Chi home practice resulting in 23.6 h of Tai Chi practice. The study sets out to achieve 50 h; therefore, adherence remains a challenge. Despite this, it is acknowledged that the concept of ‘dose’ is poorly understood and there is a lack of understanding of the specific dose of Tai Chi necessary within this population to yield a change. Further research is required to establish a dose–response relationship for Tai Chi in people with dementia.

Another possible explanation for the lack effect may lie with the lack of improvement in Tai Chi. It is highly probable that through repetitive practice, PWD will develop an enhanced capacity for actually completing the Tai Chi movements. This enhanced capacity through a combination of learning and physiological adaptation, i.e. they become better at the routines, and the muscles and movement patterns become stronger and easier. This would then ultimately carry over into enhanced function witnessed in the sub-phases of the iTUG. However, if, despite the repetitive practice, the PWD demonstrated no improvement in Tai Chi, this would suggest that this process of adaptation had not occurred, thus offering an explanation for lack of effect. No measures of ability to complete Tai Chi were taken; therefore, this remains speculative.

It may be possible that the iTUG was not the optimal measure to detect change following Tai Chi. It is possible the iTUG lacked the sensitivity and specificity to detect change following the intervention. Minimal detectable change values for the total time to complete iTUG have been established but this is not the case for the sub-phases of iTUG. Moreover changes in balance and physical functioning may have been enhanced through Tai Chi but were not captured through the measurement of iTUG. The iTUG is comprised of sub-phases each of which has a different underlying construct. For example, to demonstrate high vertical acceleration during sit to stand requires lower limb power and to turn rapidly requires, among other things, rapid asymmetrical coordination. It is possible that, through Tai Chi, with its mindful, slow, moving meditation, this training stimulus may not be best placed to enhance higher-order temporal kinematics such as velocity and acceleration. This so-called specificity principle of training is well understood and may offer an explanation as to why velocities and acceleration where unchanged [37]. Indeed, in studies investigating the effects of exercise prescribed to closely match the demands of the task, significant change has been demonstrated, in this example for turning duration [38].

It is further possible that the lack of effect witnessed is a result of a lack of statistical power due to the smaller sample size recruited than planned. This resulted in a reduction of statistical power from the planned 90% to 69% [19]. This poses the question, if the sample size was greater, would the study have been sufficiently powered to achieve statistical significance? It has often been recommended to calculate post hoc power or observed power; however, this does not provide insights beyond those observed with statistical tests [39] mainly because of the relationship between the *p* value and observed power [40]. This approach is, therefore, not recommended [40]. In clinical studies such as this, it is more important to observe the magnitude of actual change (mean difference, Table 2). These values are small with some positive, some negative and all confidence intervals crossing zero suggesting no effect cannot be ruled out [41] and the clinical benefits of the intervention, on the iTUG, were minimal, if any. The numbers provided could be used to determine effect sizes for future studies.

This is the first study to explore the effects of Tai Chi on iTUG in PWD and, thus, represents a novel contribution to the literature. It seems possible that generating the parameterisation of the timed up and go test is quick, simple and possible within an individual home environment.

### Study limitations

A number of limitations should be acknowledged. First, a pragmatic approach to chair selection was adopted as all data were collected in the individual’s home. Every attempt was made to ensure the same chair was used for follow-up but it is possible that chairs differed between individuals. This is true also for the environment in which the iTUG was completed. Again, intra-individual variability was minimised but between-individual differences were possible. Second, it is acknowledged that there was not 100% compliance with Tai Chi, especially the home practice element.

## Conclusions

This study identified that there were no differences in performance between the control group and the Tai Chi group in their ability to complete the iTUG, regardless of sub-phase. This suggests that such an intervention had no impact on physical performance of iTUG, therefore if improvements to iTUG are a clinical aim then modifications to the treatment offered in this study are required.

## Data Availability

Data can be made available on reasonable request.
